# Idiopathic Pulmonary Fibrosis: An Update on Pathogenesis

**DOI:** 10.3389/fphar.2021.797292

**Published:** 2022-01-19

**Authors:** Qianru Mei, Zhe Liu, He Zuo, Zhenhua Yang, Jing Qu

**Affiliations:** School of Basic Medicine, Tongji Medical College, Huazhong University of Science and Technology, Wuhan, China

**Keywords:** idiopathic pulmonary fibrosis, pathogenesis, alveolar epithelial cells, fibroblasts, extracellular matrix

## Abstract

Idiopathic pulmonary fibrosis (IPF) is a progressive, lethal fibrotic lung disease that occurs primarily in middle-aged and elderly adults. It is a major cause of morbidity and mortality. With an increase in life expectancy, the economic burden of IPF is expected to continuously rise in the near future. Although the exact pathophysiological mechanisms underlying IPF remain not known. Significant progress has been made in our understanding of the pathogenesis of this devastating disease in last decade. The current paradigm assumes that IPF results from sustained or repetitive lung epithelial injury and subsequent activation of fibroblasts and myofibroblast differentiation. Persistent myofibroblast phenotype contributes to excessive deposition of the extracellular matrix (ECM) and aberrant lung repair, leading to tissue scar formation, distortion of the alveolar structure, and irreversible loss of lung function. Treatments of patients with IPF by pirfenidone and nintedanib have shown significant reduction of lung function decline and slowing of disease progression in patients with IPF. However, these drugs do not cure the disease. In this review, we discuss recent advances on the pathogenesis of IPF and highlight the development of novel therapeutic strategies against the disease.

## Introduction

Idiopathic pulmonary fibrosis (IPF) is a chronic and progressive interstitial lung disease of unknown etiology and with a poor prognosis. IPF primarily occurs in middle-aged and elderly adults. In the United States, median age of newly diagnosed patients is 62 years, and 54% of them are male (Mortimer et al., 2020). The epidemiological survey of IPF shows that the global incidence and prevalence of IPF are in the range of 0.09 and 1.30 per 10,000 people and increasing year by year. Compared with other countries studied, the United States, South Korea and Canada have the highest incidence (Schafer et al., 2020; Maher et al., 2021a). Histopathological characteristics of IPF include excessive deposition of the extracellular matrix (ECM), leading to distortion of normal lung architecture and irreversible loss of lung function (Glass et al., 2020). IPF is clinically manifested by progressive dyspnea and a significant decrease in lung compliance (Schafer et al., 2020). In the past decade, significant progress has been made on our understanding of the mechanisms underlying this disease. The development of IPF is thought to be associated with both genetic and environmental factors. It is proposed that repetitive micro-injuries to alveolar epithelial cells trigger abnormal epithelial-fibroblast communication, which eventually results in abnormal ECM accumulation and pathological lung remodeling (Heukels et al., 2019; Martinez et al., 2017; Richeldi et al., 2017).

**FIGURE 1:**
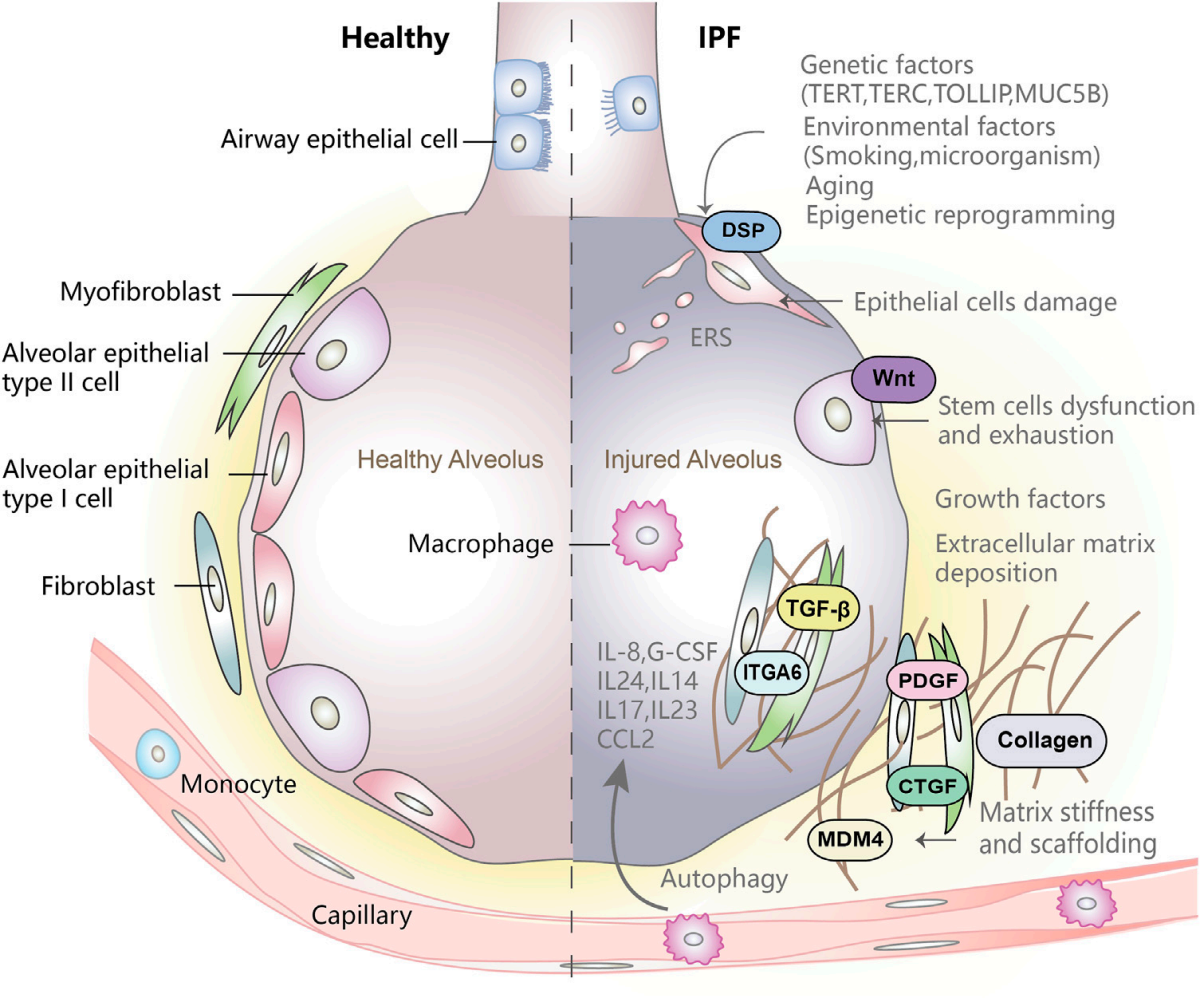
Pathogenesis of idiopathic pulmonary fibrosis. Genetic factors affect the integrity of epithelial cells, environmental factors and aging-related changes will trigger epigenetic reprogramming. The combined action of the three factors will cause epithelial cell damage and trigger the abnormal activation of epithelial cells. Activated epithelial cells secretes a large number of cytokines such as TGF-β which consequently promotes fibroblast migration and proliferation, and also promote fibroblasts to differentiate into myofibroblasts. Myofibroblasts secrete large amounts of ECM, leading to ECM deposition. In addition, epithelial cell damage, disfunction and exhaustion of stem cells, abnormal deposition of extracellular matrix and matrix stiffness play a vital role in progression of abnormal lung fibrosis and remodeling of lung structure.

There is a growing portfolio of treatment options for IPF. Two drugs, nintedanib and pirfenidone, have been approved for treatment of patients with IPF. Nintedanib is a tyrosine kinase inhibitor, while pirfenidone is an oral pyridine that has anti-inflammatory, antioxidant, and anti-fibrotic effects. The two drugs have demonstrated reduction of lung function decline and slowing of disease progression, but they were also associated with some side effects and tolerability issues (Liu et al., 2017). Lung transplantation is the fundamental treatment for IPF. The average survival time of post-transplantation is 4–5 years. However, due to the restricted supply of donor organs and the limitation of chronic allograft rejection, only a few patients can receive this intervention (Kumar et al., 2018). Currently, IPF management is still aim to ameliorate symptoms, improve health status and preserve lung function (Glassberg, 2019). A better understanding of the pathogenesis of IPF will benefit the development of more efficient and safer therapies against IPF. This review will summarize recent advances in the pathogenesis of IPF (Figure 1) and highlight promising novel therapeutic strategies against the devastating fibrotic lung disease.

## Risk Factors

### Genetic

Genetic factors play an important role in the development of IPF (Figure 1). Genetic susceptibility of IPF includes single nucleotide polymorphisms (SNPs) and the resultant changes in gene expression. Familial interstitial pneumonia (FIP) is an autosomal dominant genetic disease with variable penetrance in which rare genetic variants have been identified (Kropski et al, 2015; Lorenzo-Salazar et al., 2019). These genetic variations involve the maintenance of telomere length (telomerase reverse transcriptase-TERT, telomerase RNA component-TERC, poly (A) -specific ribonuclease-PARN and regulator of telomere elongation helicase-RTEL) (Barros et al., 2019) and epithelial barrier function (desmoplakin-DSP, dipeptidyl peptidase 9-DPP9, AKAP13, CTNNA) (Allen et al., 2017; Fingerlin et al., 2013). Mutations in Toll-interacting protein (TOLLIP) (encoding an inhibitor of transforming growth factor-β (TGF-β) pathway and key regulator of Toll-like receptor-mediated innate immune responses) are associated with decreased expression of TLR mRNA and increased susceptibility to lung infections (Barros et al., 2019).

The genome-wide association studies (GWAS) found that a SNP (rs35705950) in the promoter region of mucin 5B (MUC5B) greatly increases the risk of IPF (Moore et al., 2019). MUC5B contributes to airway mucus production and plays an important role in innate immunity of lungs. Overexpression of MUC5B is related to impaired mucociliary clearance (MCC) and the degree and duration of fibrosis (Hancock et al., 2018; Zhang Q et al., 2019). The rs35705950 minor allele mutation can lead to overexpression of mucin 5B in small airway epithelial cells, and DNA methylation is closely related to genetic susceptibility of MUC5B (Zhang Q et al., 2019). In addition, a study identified a positive feedback bistable ERN2-XBP1S pathway upregulated MUC5B mRNAs in IPF and further regulated mucus secretion, providing an unfolded protein response (UPR)-dependent mechanism with rs35705950 variant (Chen G et al., 2019).

### Environmental

Environmental exposure and genetic predisposition may have a synergistic effect in the development of IPF (Figure 1). In both sporadic and familial pulmonary fibrosis, environmental exposures to lung epithelium can increase the risk of IPF. Of them, smoking and metal dust are the strongest risk factors (Kc et al., 2018; Pardo and Selman, 2020). Cigarette smoke can cause a variety of cellular changes through epigenetic mechanisms. It also induces miRNA imbalance and ER stress, promoting spontaneous lung injury and differentiation from fibroblast to myofibroblast (Mascaux et al., 2009; Song et al., 2019). Pollutants and ultrafine particles in cigarette smoke contain carbon black (CB) and cadmium (Cd). The content of Cd and CB in IPF lung tissue increased significantly and was directly proportional to the amounts of citrullinated vimentin (Cit-Vim). Under activation of Akt1 and peptidylarginine deiminase 2 (PAD2), Cd/CB can induce Vim citrullination and Cit-Vim secretion, which in turn triggers fibroblasts to infiltrate lung microspheres, promotes increased expression of collagen and α-smooth muscle actin (α-SMA), and induces lung fibrosis (Li et al., 2021).

Microorganisms (viruses, fungi and bacteria) play a potential role in the pathogenesis of IPF (Lipinski et al., 2020). Compared with normal people, IPF patients have an imbalance in the composition of the lung microbiota, which can serve as a persistent stimuli for repetitive alveolar injury (Molyneaux et al., 2017). The inflammatory and fibrotic mediators and immune disorders in the lungs of IPF patients are related to bacterial load. In animal models of pulmonary fibrosis, pulmonary dysbiosis precedes the peak of lung injury and persists throughout the period of fibrosis. After adjusting the relevant clinical and physiological variables, lung bacterial burden can predict disease progression of IPF patients (O'Dwyer et al., 2019). In addition, Epstein-Barr virus (EBV), cytomegalovirus (CMV) and human herpes virus are detected in alveolar epithelial cells of patients with IPF, suggesting a link between viral infection and increased risk of IPF. Although the mechanisms by which viral infection is associated with IPF remains unclear, studies suggest that it may be related to activation of epithelial-mesenchymal transition, promotion of TGF-β expression, and induction of epigenetic reprogramming (Li et al., 2018; Sides et al., 2011). Interestingly, IPF patients expressing MUC5B risk alleles have a significantly lower bacterial burden compared with the patients who do not bear the risk allele (Molyneaux et al., 2014). NEDD4-2 modulates epithelial Na^+^ channel (ENaC) through ubiquitination, which is essential for proper mucociliary clearance of inhaled irritants and pathogens. Recent study has shown that expression of NEDD4-2 is reduced in IPF lung tissue. NEDD4-2 promotes fibrosis remodeling through regulating the expression of proSP-C, Smad2/3 and TGF-β signaling pathway (Duerr et al., 2020).

### Aging

Aging is a pathological feature of both human IPF and experimental lung fibrosis in animals. The major characteristics of aging lungs include telomere mutations, epigenetic changes, loss of protein homeostasis, mitochondrial dysfunction, and cellular senescence (Figure 1). Telomere mutations often result in abnormal DNA repair and genome instability, which serves as a trigger for cell senescence (Lopez-Otin et al., 2013; Barros et al., 2019). In addition to DNA damage, telomere shortening or damage may also promote fibrosis by impairing tissue repair function, activating p53, reducing mitochondrial biosynthesis, and triggering cellular senescence pathways (Sui et al., 2016; Pineiro-Hermida et al., 2020). There is evidence that in IPF, most of the changes related to aging, including shortening of telomeres which mainly occurs in Alveolar epithelial type II cells (AT2) (Selman et al., 2019). F-box and WD repeat domain-containing protein 7 (FBW7) is identified as a driver of pulmonary premature senescence and fibrosis. It is an E3 ubiquitin ligase that facilitates telomere protective protein 1 (TPP1) multisite polyubiquitination and accelerates degradation through binding to TPP1, thereby triggering telomere uncapping, cause cell senescence and tissue fibrosis (Wang L et al., 2020). Studies have shown that expression of core senescence-related markers is significantly elevated in IPF AT2 cells. These markers include CDKN1A/p21, CDKN2A/p16, TP53, MDM2, CCND1 (Barnes et al., 2019; Liu B et al., 2019). In addition, aging may cause dysfunction of stem/progenitor cell renewal, rendering alveolar epithelial cells incapable of repairing and regenerating injured lungs. Aging epithelial cells can produce a variety of pro-inflammatory and pro-fibrotic mediators, such as Interleukin 6 (IL-6), IL-1 and TGF-β, which are part of the senescence-associated secretory phenotype (SASP) (Merkt et al., 2020). In contrast, aging fibroblasts have stronger anti-apoptotic ability to resist environmental stress and can increase extracellular matrix components (Mora et al., 2017; Moore et al., 2019). Metabolic changes, such as glycolytic reprogramming, also play an important role in the pathogenesis of pulmonary fibrosis. Human metabolomics studies have shown that IPF lung tissues displayed increased glycolysis compared with healthy controls (Zhao et al., 2017). Specifically, aging fibroblasts increase glucose utilization and increase resistance to apoptosis (Cho et al., 2017; Selvarajah et al., 2019; Cho and Stout-Delgado, 2020). Studies have suggested that plasminogen activator inhibitor 1 (PAI-1) can protect (myo) fibroblasts from apoptosis in old mice. PAI-1 is the effector molecule of TGF-β, which can induce senescence by inducing p21 (Huang et al., 2015). Aged mice develop nonresolving pulmonary fibrosis following lung injury. Intriguingly, p53 signaling is abnormally activated in aging AT2, and silencing the expression of p53 can inhibit the development of progressive fibrosis (Borok et al., 2020; Yao et al., 2020). Changfu Y et al. determined that senescence rather than AT2 cell depletion is the key link in promoting progressive fibrosis, so genetic intervention for p53 activation and senescence will become a therapeutic target for pulmonary fibrosis in the future (Yao et al., 2020). These findings suggest that targeting aged cells may be effective for the treatment of fibrotic lung disease.

### Epigenetic Reprogramming

Increasing evidence demonstrated that under the influence of environmental factors and aging, epigenetic changes play an important role in IPF (Kadel et al., 2019; Yamada, 2020; Yang and Schwartz, 2015). Epigenetic modifications include DNA methylation, histone modifications, and changes in the expression of non-coding RNA (especially microRNA) (Chen et al., 2017). When individuals are exposed to environmental stresses, such as smoke and dust, air pollution can cause epigenetic changes. It has been shown that silica exposure increases expression of DNA methyltransferase 1 (DNMT1) in patients with IPF, leading to the accumulation of collagen and lung fibrosis (Zhang N et al., 2019).

Genome-wide methylation analysis has shown that there are 2130 differentially methylated regions (DMR) in IPF lungs compared with healthy lungs (Yang et al., 2014). Methylation in these DMRs may regulate expression of multiple target genes and miRNAs involved in the development of IPF (Luo et al., 2020). Changes in DNA methylation correspond to altered mRNA expression of a variety of genes, some of which, such as apoptosis regulation and biosynthesis processes, have known roles in IPF (Cisneros et al., 2012; Rabinovich et al., 2012; Sanders et al., 2012). Regulation of DNA methylation has been demonstrated as a key pathogenetic pathway in TGF-β-induced lung fibrosis, through reduction of prostaglandin E2 (PGE2) and stimulation of epithelial-mesenchymal transition (Hu et al., 2010; Huan et al., 2015). Li et al. confirmed that MBD2 is highly expressed in macrophages in fibrotic lung. MBD2 is a member of the methyl-CpG-binding domain (MBD) proteins family. It directly binds to methylated CpG DNA to regulate PI3K/Akt signaling, thereby enhancing macrophage M2 program and promoting TGF-β signaling (Wang et al., 2021). In addition, HMG AT-hook 2 protein (HMGA2), a member of high-mobility group (HMG) proteins family, can regulate the transcription of its target genes by changing the chromatin structure at the promoter and/or enhancers, which can mediate transformation TGF-β1 signaling. Recent studies have found that inhibiting HMGA2-FACT-ATM-pH2A.*X* axis of human lung fibroblasts *in vitro* could reduce fibrotic hallmarks (Dobersch et al., 2021).

Studies of histone modifications of IPF lungs found that when treated with histone modification-related drugs (TSA, SpA, etc.), the level of surfactant protein C (Sp-C), the activation and proliferation of fibroblasts are all significantly affected. Additionally, histone modifications can also affect the anti-apoptotic ability of fibroblasts by inhibiting anti-fibrotic genes such as FAS and caveolin 1 (Cav-1) (Bartczak et al., 2020). Ligresti,G et al. identified that histone methyltransferase G9a/chromobox homolog 5 (CBX5)/methylated lysine 9 residue on histone 3 (H3K9me) were key regulators of fibroblast activation, and determined that by participating in the CBX5/G9a pathway, TGFβ and increased matrix stiffness effectively inhibited PGC1α expression in lung fibroblasts (Ligresti et al., 2019).

miRNA microarray analysis showed that expression of miR-21 and miR-199a-5p was increased in IPF lungs, while expression of miR-31, let-7 and miR-200 was decreased (Yang et al., 2015). Among them, miR-21 can induce epithelial-mesenchymal transition (EMT) by inhibiting Smad7 and promote TGF-β-induced fibrosis (Liu et al., 2010), while Let7 may participate in fibrotic process by targeting HMGA2 (Chirshev et al., 2019). The miR-200 family promote AT2s to restore its ability to transdifferentiate into Alveolar epithelial type I cells (AT1) (Moimas et al., 2019). The presence of miR-145 can activate TGF-β to induce fibrosis, and it can also induce expression of α-smooth muscle actin (α-SMA), which is a hallmark of fibroblast to myofibroblast differentiation (Yang et al., 2013). MiR-424 targets Smurf2 (TGF-β pathway inhibitor) to promote fibroblast differentiation and promote TGF-β secretion (Xiao et al., 2015). MiR-301a can regulate fibroblast activation induced by TGF-β and IL-6 (Wang J et al., 2020). miR-29 is a main negative regulator of ECM production. Further studies have found that IPF ECM inhibited miR-29 expression upstream at the transcriptional level, and suppressed Dicer1 downstream at the processing steps to maintain the fibrosis progression (Herrera et al., 2018).

## Cells and Regulators

### Epithelial Cells Damage

Currently, the prominent initiation of IPF is widely considered to be repeated lung epithelial cell damage and repair dysfunction. Under normal circumstances, the damage of alveolar epithelial cells will lead to recruitment of inflammatory cells, fibrosis and matrix deposition in order to repair the damaged cells. This stage is temporary and then normal pulmonary homeostasis will be restored through activation of apoptotic pathways and phagocytosis of macrophages during the injury repair stage (Desmouliere et al., 1995). However, in IPF lungs, mutations of lung epithelial restriction genes (SFTPC, SFTPA2 and ABCA3) and abnormal expression of genes such as MUC5B cause lung epithelial mucosal barrier dysfunction (Hancock et al., 2018). Repeated stimulation of microorganisms, smoking, gastroesophageal reflux and other factors destroys the integrity of the lung epithelium. In addition, inflammation, excessive production of reactive oxygen species (ROS) and endoplasmic reticulum stress (ERS) in IPF lungs lead to repetitive damage to epithelial cells (Ornatowski et al., 2020).

In the past decades, there has been numerous works to determine the central role of stem cells in epithelial repair, including AT2s and its subsets, basal cells, bronchoalveolar alveolar stem cells (BASC) (Frank et al., 2016; Hewlett et al., 2018). Studies have identified a subset of fibroblasts expressing PDGFRα^+^ or Lgr5^+^ can participate in alveolar homeostasis by stimulating Wnt signaling (Axin2+) located in the alveolar compartment. These subset of fibroblasts are involved in promotion of alveolar growth and maturation, and preferentially differentiate into myofibroblasts after lung epithelial injury (Lee et al., 2017; Zepp et al., 2017).

### Lung Stem Cells Dysfunction and Exhaustion

In IPF, genetic and environmental factors may cause damage to AT1s, while dysfunction of AT2s makes it difficult to repair the damaged AT1s. AT2s, serve as the predominant epithelial progenitor in alveoli, play an important role in maintaining lung homeostasis (Parimon et al., 2020). Abnormal function of alveolar epithelial cells is associated with activation of signal pathways such as Wnt/β-catenin and Sonic Hedgehog (Stewart et al., 2003; Nabhan et al., 2018; Reyfman et al., 2019). Recent study identified a rare subset of mature AT2 cells with stem cell propertie marked by continuous expression of the Wnt target gene Axin2. Canonical Wnt signaling pathway blocks reprogramming of alveolar stem cells into AT1 cells. When injury occurs in epithelial cells, Wnt signaling pathway is activated and participates in " ancillary” AT2 stem cell progenitor cell activity (Nabhan et al., 2018). Wnt-reactive alveolar epithelial progenitor cells (AEP) in AT2 are a stable lineage during alveolar homeostasis, but rapidly expand to regenerate most of the alveolar epithelium after acute lung injury, showing stronger “stemness”. AEPs have a unique transcriptome, epigenome and functional phenotype, and specifically respond to Wnt and Fgf signaling (Zacharias et al., 2018). It has been believed that repetitive micro-injuries are a potential cause of AT2 depletion. However, the reduction of AT2 number in IPF lung supports the idea of stem cell exhaustion. In addition, aging, ERS and mitochondrial dysfunction play an important role in AT2 depletion and impaired self-renewal (Kropski and Blackwell, 2018; Borok et al., 2020; Parimon et al., 2020). Loss of Cdc42 in AT2s results in impaired differentiation, exposing alveolar cells to sustained elevated mechanical tension which activates a TGF-β signaling loop in AT2 cells in a spatially regulated manner, thereby promoting lung fibrosis progression from periphery to center (Wu et al., 2020).

### Fibroblasts and Myofibroblasts

In IPF, pro-fibrotic mediators secreted by activated fibroblasts continue to act on fibroblasts to form a positive feedback, which leads to production of ECM and myofibroblast differentiation (Wipff et al., 2007). TGF-β is considered to be the primary factor that promotes fibroblast differentiation into myofibroblasts (Huang et al., 2020). Myofibroblasts secrete more ECM than fibroblasts. They are the main collagen-producing cells in the lung and are characterized by expression of contractile protein α-SMA and fibroblast activation protein (FAP) (Tsukui et al., 2020). FAP is a membrane-spanning protein that is essential for collagen remodeling. As FAP exhibits a low expression state in most healthy cells, it can be used as a molecular marker to exploit for specifically target drugs to fibroblasts that cause fibrosis (Hettiarachchi et al., 2020).

In the normal wound healing process, unwanted fibroblasts are eliminated by activating the apoptotic pathway. The elimination mechanism of fibroblasts limits the ongoing matrix deposition and fibrosis (Desmouliere et al., 1995). In IPF, myofibroblasts were found to resist FAS ligand-induced apoptosis and have stronger proliferation ability when grown on polymerized collagen (Xia et al., 2008; Nho et al., 2011). FasL, Tumor necrosis factor (TNF)-related apoptosis-inducing ligand (TRAIL) and Cav-1 protein expression in these cells decreased, while AKT activity increased (Hohmann et al., 2019). In addition, myofibroblast contraction is irreversible, which contribute to regulate the remodeling of collagen, trigger the spatial structural reorganization of collagen fibrils, increase their mechanical stress, and stiffen the ECM (Zhou et al., 2020). Periostin is highly expressed in IPF lungs. Further studies have found that in lung fibroblasts, periostin/integrin αVβ3 can promote expression of cell cycle-related molecules, including cyclins, cyclin-dependent kinases (CDKs) and E2F families, and transcription factors (such as B-MYB and FOXM1), which play a vital role in the proliferation of lung fibroblasts (Yoshihara et al., 2020).

### Basal Cells

An important feature of epithelial cell remodeling in IPF is the expansion of distal basal cells, which can serve as stem/progenitor cells of the pseudostratified epithelium of the lung. In the cellular area of IPF, secretory sensitized basal cells (SPB) are enriched, and the formation and secretion function of its subpopulations are regulated by Notch signal. Specifically, NOTCH2 restricts the differentiation of basal cells, while NOTCH3 can inhibit secretory differentiation (Carraro et al., 2020). In addition, RNA-seq analysis for IPF indicates that expression of LncRNA MEG3 in basal cells increased. MEG3 plays a role in abnormal epithelial cell differentiation in IPF and regulates epithelial cell migration related genes including TP63, STAT3, KRT14, YAP1 and AXL, which together contribute to the restructuring of IPF (Gokey et al., 2018). Milena S et al. confirmed that MMP9 expressed by airway base cells (ABC) in IPF was significantly increased and regulated by TGF-β pathway. When targeting MMP9, the anti-fibrotic effect is related to the reduction of TGF-β activation in a subgroup of IPF patients, which reveals an association with expression of type 1 IFN in ABC-like cells (Espindola et al., 2021).

### Growth Factors

There is overwhelming evidence in support of a key role of TGF-β in the pathogenesis of IPF (Huang et al., 2020). TGF-β promotes epithelial-mesenchymal transition, epithelial cell migration, fibroblast proliferation, activation, and differentiation into myofibroblasts (Figure 1). TGF-β can also increase the production of other fibrotic mediators and pro-angiogenic mediators (Grimminger et al., 2015; Song et al., 2020). TGF-β is synthesized as a latent complex by binding to the latency-related peptide (LAP), which covalently binds to the ECM protein (Biernacka et al., 2011). Latent TGF-β can be activated by a range of factors, including ανβ6 integrin (John et al., 2020). In IPF, expression of ανβ6 in alveolar epithelial cells is increased, which binds to LAP to induce TGF-β activation (Jenkins et al., 2006). Once activated, TGF-β binds to its receptors and stimulates phosphorylation of transcription factor Smad3. Phospho-Smad3 interacts with Smad4 to form a complex which translocates into the nucleus to induce expression of target genes, including profibrotic genes such as α-SMA, CTGF and ECM major collagen 1A1 (COL1A1) (Biernacka et al., 2011; Massague, 2012). Interestingly, the expression of negative-regulating factor tripartite motif 33 (TRIM33) of TGF-β/SMAD in IPF increased. TRIM33 is an E3 ubiquitin ligase, which can promote SMAD4 ubiquitination and induce SMAD4 to export from the nucleus, thereby inhibiting transcriptional activity of SMADs. However, the combination of TRIM33 and small heat shock protein (HSPBS) weakened its inhibitory activity. The upregulation of TRIM33 may be regarded as a failed attempt to prevent the progression of fibrosis in IPF and lung fibrosis models (Boutanquoi et al., 2020).

CTGF, also known as cellular communication network factor 2 (CCN2), is an important mediator of organ fibrosis in human body (Falke et al., 2020; Wang et al., 2019). It is considered to be a predictor of pulmonary fibrosis disease and a potential target for anti-fibrosis therapy (Leask, 2011). CTGF is secreted and activated under stimulation of TGF-β. CTGF mediates lung matrix deposition and fibroblast differentiation by activating downstream MAP kinase pathway (Duncan et al., 1999; Inui et al., 2021). In addition, CXCL12 can also induce the expression of CTGF in human lung fibroblasts by activating the MEKK1/JNK signaling pathway (Lin et al., 2018). Studies have found that gene promoter of CTGF contains numerous transcription factor binding sites such as NF-κB, signal transducer and activator of transcription (STAT), activator protein-1 (AP-1) and SMAD (Lin et al., 2018), indicating these factors may affect IPF through CTGF.

PDGF is widely expressed in macrophages, platelets, endothelial cells and fibroblasts (Hewlett et al., 2018). Highly expressed PDGF can be detected in BALF of IPF patients and bleomycin-induced IPF model mice (Phan et al., 2021). The abnormal expression and signal transduction of PDGF ligands and receptors have been confirmed to be closely related to IPF. In IPF, TGF-β signaling promotes expression of PDGF-B through regulatory T cells (Tregs), thereby stimulating PDGF-B-mediated fibroblast proliferation and migration (Kanaan and Strange, 2017; Kishi et al., 2018).

Insulin-like growth factor (IGF1) is a key molecule that regulates cellular senescence (Duran-Ortiz et al., 2021). As mentioned above, senescence has been identified as an important reason for the weakened repair function of AT2s in IPF. Under pathological conditions, ATs release IGF1, which activates the surface of adjacent normal ATs. IGF receptor (IGFR-1), and further activate the PI3K/AKT signaling pathway, and participate in ATs senescence and IPF by releasing CTGF, TGF-β1 and MMP9 (Sun et al., 2021).

## Molecular Mechanisms

### Extracellular Matrix Deposition

The massive deposition of extracellular matrix in IPF is mainly involved in changes in two families of proteins: MMPs and tissue inhibitors of metalloproteinases (TIMPs) (Figure 1). Studies have found that expression levels and localization of MMP and TIMP in IPF lungs undergo substantial changes. The levels of MMP1, MMP2, MMP9 and the four TIMPs are up-regulated. Among them, MMP1 is more common in alveolar macrophages and epithelial cells, while TIMP is highly expressed by myofibroblasts in IPF fibroblastic foci (Betensley et al., 2016). The extracellular matrix of IPF can also change transcriptional profile of lung fibroblasts and affect the translation of ECM proteins, such as COL1A1, COL1A2, COL3A1, COL5A2, COL4A2, MMP2, MMP3, MMP10 and TIMP2 (Zolak and de Andrade, 2012). Together, these findings suggest that there is a positive feedback pathway between fibroblasts and abnormal ECM, in which the fibrotic extracellular matrix is both the cause and the result of fibroblast activation (Guiot et al., 2017).

### Matrix Stiffness and Scaffolding

Matrix stiffening is a prominent feature of lung fibrosis. Compared with healthy lung scaffolds, IPF scaffolds increase tissue stiffness, density, ultimate force, and differential expressions of matrisome proteins (Figure 1). The collagen, proteoglycan and ECM glycoprotein in the IPF scaffold increased, but specific basement membrane (BM) proteins (such as laminins and collagen IV) were decreased, while nidogen-2 was increased, accompanied by periostin and proteoglycans production were increased (Elowsson Rendin et al., 2019). The increased stiffness of ECM tissue is a result of dysregulated collagen cross-linking, which is related to post-translational modification of collagen involved in lysyl oxidase-like (LOXL) 2 and LOXL3 (Jones et al., 2018). ECM stiffness participates in the pathogenesis of IPF. Accumulating evidence indicates that mechanical interactions between fibroblasts and the stiffened ECM provide a feedforward mechanism that sustains and/or perpetuates pulmonary fibrosis (Zhou et al., 2013).

In previous study, we demonstrated that matrix stiffness regulates the ability of fibrotic lung myofibroblasts to invade the BM, by increasing α6-expression, mediating MMP-2-dependent pericellular proteolysis of BM collagen IV. Genetic ablation of α6 in collagen-expressing mesenchymal cells or pharmacological blockade of matrix stiffness-regulated α6-expression protects mice against bleomycin injury-induced experimental lung fibrosis. Studies found that a mechanotransduction pathway involving Rho/Rho kinase (Rho/ROCK), actin cytoskeletal remodeling, and a mechanosensitive transcription factor, megakaryoblastic leukemia 1 (MKL1), that coordinately regulate myofibroblast differentiation, and pharmacologic disruption of this pathway with the ROCK inhibitor fasudil induced myofibroblast apoptosis through a mechanism involving downregulation of BCL-2 and activation of the intrinsic mitochondrial apoptotic pathway (Zhou et al., 2013). Recently, we have shown that mouse double minute 4 homolog (MDM4) is a matrix stiffness-regulated endogenous inhibitor of p53. MDM4 is highly expressed in the fibrotic lesions of human IPF and experimental pulmonary fibrosis in aged mice. Our studies provides evidence that mechanosensitive MDM4 is a molecular target with promising therapeutic potential against persistent lung fibrosis associated with aging (Qu et al., 2021). Moreover, ECM stiffness is sensitive to exogenous TGF-β stimulation through inhibiting the interaction of inner nuclear membrane protein LEM domain-containing protein 3 (LEMD3) and SMAD2/3. LEMD3 is physically connected to the actin cytoskeleton of cells and inhibits TGF-β signaling (Chambers et al., 2018). At the metabolic level, increased matrix stiffness impairs the synthesis of anti-fibrotic lipid mediator PGE2 and reduces expression of rate-limiting prostaglandin biosynthetic enzyme cyclooxygenase 2 (COX-2) and prostaglandin E synthesis (PTGES) through p38/MAPK signaling pathway (Berhan et al., 2020). Genome-wide association studies (GWASs) have identified DSP (desmoplakin) gene, a type of intercellular junction responsible for maintaining the structural integrity and mechanical stability of the epithelium, as a significant locus associated with IPF (Fingerlin et al., 2013; Mathai et al., 2016; Allen et al., 2017; Tasha E). Our studies demonstrated that matrix stiffness regulates DSP gene expression by an epigenetic mechanism involving alteration of DNA methylation in the DSP promoter. Targeted DNA methylation by CRISPR (clustered regularly interspaced short palindrom2076295ic repeats)/dCas9 (deactivated CRISPR-associated protein-9 nuclease)-mediated epigenome editing effectively reverses stiff matrix-induced DSP overexpression (Qu et al., 2018). We speculate that aberrant DSP expression in IPF may not only represent a robust and persistent epithelial response to chronic/repetitive lung injury but also actively participate in aberrant lung repair and/or the restoration of lung epithelial function (Qu et al., 2018). In addition, studies have identified that rs2076295 (an intron variant in DSP gene) was related to IPF susceptibility and directly regulated DSP expression in human airway epithelial cells. Deletion of DSP enhances expression of extracellular matrix-related genes such as matrix metalloproteinases 7 (MMP7) and MMP9 and promotes cell migration (Hao et al., 2020). For IPF patients with DSP alleles and MUC5B alleles, the mortality rate is lower, and anti-fibrosis drugs are more effective in treatment (Doubkova et al., 2021). These studies indicate that targeting mechanosensitive signaling in myofibroblasts may be an effective approach for treatment of fibrotic disorders.

### Endoplasmic Reticulum Stress

Endoplasmic reticulum stress (ERS) occurs when there is an imbalance between cell’s demand for protein synthesis and the ability of endoplasmic reticulum to synthesize, process, and package proteins. As ERS occurs, cells activate an UPR, which attempts to restore normal function of the endoplasmic reticulum. When ERS is persisting or severe, it triggers cell apoptosis (Kropski and Blackwell, 2018; Hipp et al., 2019). It has been observed that markers of UPR activation in AT2 in IPF patients are elevated (Kropski and Blackwell, 2018; Baek et al., 2020). ERS may synergize with inflammation and viral infection to induce epithelial cell damage (Chen T et al., 2019). In IPF, UPR stimulates the production of fibrotic mediators, such as TGF-β, PDGF (platelet-derived growth factor), CXCL12 (CXC chemokine 12), CCL2 (chemokine CC ligand 2) (Wolters et al., 2014; Kropski and Blackwell, 2018). The chaperone protein GRP78 (glucose regulatory protein 78) is the main regulator of ER homeostasis and suppresses UPR by interacting with transmembrane ER stress sensors. It is found that the expression of GRP78 in AT2 cells from old mice and IPF lungs decreased, while GRP78 knocked out will induce ERS, apoptosis and lung inflammation to promote fibrosis (Borok et al., 2020). Otherwise, thioredoxin domain containing 5 (TXNDC5), an ER protein enriched in fibroblasts, is highly up-regulated in fibroblasts from IPF lung/BLM-induced mouse and enhances TGF-β signaling by increasing and stabilizing TGF-beta receptor 1 (TGFBR1), while TGF-β promotes TXNDC5 expression via ATF6 ER stress pathway, forming a positive feedback loop (Lee et al., 2020). Mutations in the genes encoding surfactant proteins [surfactant protein C (SFTPC) and A2 (SFTPA2)] can lead to abnormal surfactant folding and ERS, and promote epithelial-mesenchymal transition (Mulugeta et al., 2015; Takezaki et al., 2019).

### Inflammation and Immunity

The role of inflammation in the development of IPF remains controversial. In the early stage of alveolar injury, neutrophils are recruited into the injured sites, triggering an immune response by releasing pro-inflammatory cytokines and producing neutrophil elastase (NE) to exacerbate fibrosis (Le Saux and Chapman, 2018). Elevated IL-8 and G-CSF have been found in the bronchoalveolar lavage fluid (BALF) and sputum of IPF patients, suggestive of infiltration and activation of neutrophils (Figure 1). IL-8 promotes the development of fibrosis through elastase-mediated activation of TGF-β (Betensley et al., 2016; Guiot et al., 2017; Heukels et al., 2019). IL-24 and IL-4 can synergistically induce M2 program of macrophages, thereby promoting the development of lung fibrosis (Rao et al., 2021). IL17 secreted by Th17 cells can directly promote fibrosis. In acute exacerbation of pulmonary fibrosis, the levels of IL17 and IL23 are increased, and treatment with interleukin-23 antibody can significantly attenuate airway inflammation and fibrosis and reduce IL17 level, suggesting IL23 is essential for the development of acute exacerbation of pulmonary fibrosis (Senoo et al., 2021). Monocyte and macrophages drive fibrosis through excessive repair responses to alveolar cell injury. Compared with normal lungs, the subpopulation of macrophages that highly express SPP1 and MERTK (SPP1hi) and NTN1 (laminin-like protein netrin-1) increased significantly in IPF lungs. The highly proliferated SPP1hi macrophages upregulate the expression of type 1 collagen and MMP2 and contribute to tissue repair and fibrosis (Morse et al., 2019). It has been reported that macrophage-derived NTN1 drive the development of fibrosis through a mechanism involving remodeled adrenergic nerves and their secretory product noradrenaline and α_1_ adrenoreceptors (Gao et al., 2021). CCL2 and colony stimulating factor (M-CSF/CSF1) derived from monocytes/macrophages may have a direct fibrotic effect (Coward et al., 2010). Furthermore, recent studies have identified that immune cells in lung tissue predict the severity of IPF and participate in the progress of this disease, which can be used as a reference indicator (Wang Z et al., 2020).

### Autophagy

Autophagic pathways (including macroautophagy and mitophagy) in IPF lung epithelial cells and fibroblasts are reduced, aggravating inflammation and fibrosis (Racanelli et al., 2018). Autophagy is involved in the regulation of ECM formation (Lin and Xu, 2020). Studies have shown that increasing autophagy clearance of type 1 collagen in lung fibroblasts can reduce invasiveness of IPF fibroblasts (Surolia et al., 2019). In addition, expression of the autophagy marker LC3B has been found to be significantly reduced in IPF lung fibroblasts (Ghavami et al., 2018). Specifically, the Akt signal pathway directly acts on FoxO3a to reduce its expression, further inhibiting production of autophagy marker LC3B on the collagen matrix, leading to excessive collagen accumulation (Im et al., 2015).

## Treatments

Recent research on pathogenesis of IPF has promoted notable advances in pharmacotherapeutic treatment. There are currently two recommended antifibrotic drugs, nintedanib and pirfenidone, have been shown to delay the progression of pulmonary fibrosis and reduce mortality, but there is still no cure for IPF (Cerri et al., 2019; Somogyi et al., 2019). Therefore, new treatment methods and drug targets are needed. Here we summarize some important novel drugs that have been tested in phase II-III trails (Table 1). The potential molecular targets of the drugs are also discussed. In addition, strategies of non-pharmacological treatment such as symptomatic support therapy, lung transplantation, comorbidities and management of acute exacerbation of IPF (AE-IPF) are believed to improve symptom control and quality of life (Caminati et al., 2019).

**TABLE 1:** Drugs used in the treatment of Idiopathic pulmonary fibrosis (IPF).

Drugs	Mechanism of action	References
Pirfenidone	Anti-fibrotic drug	Nathan et al. (2019)
Nintedanib	Anti-PDGFR, VEGFR, FGFR drug	Flaherty et al. (2019), Makino (2021)
Pamrevlumab	Anti-CTGF antibody	Richeldi et al. (2020)
GSK3008348	αvβ6 antagonist	John et al. (2020)
sildenafil	phosphodiesterase-5 inhibitor	Kang & Song, (2021)
Co-trimoxazole or doxycycline	Antimicrobial drug	Wilson et al. (2020)
Lebrikizumab	Anti-IL13	Maher et al. (2021b)
Carlumab	Anti-CCL2	Raghu et al. (2015)
Simtuzumab	Anti-LOX antibody	Raghu et al. (2017)

### Pirfenidone

Pirfenidone (PFD) is a pharmacological compound for IPF treatment (Flaherty et al., 2019). PFD treatment can reduce all-cause mortality and risk of hospitalization, and benefit patients with advanced pulmonary fibrosis (Nathan et al., 2019). The mechanism of PFD treating IPF is currently unclear. PFD can inhibit TGF-β-mediated fibroblast proliferation and differentiation of fibroblasts into myofibroblasts by attenuating signal transduction induced by TGF- β1/Smad3 (Molina-Molina et al., 2018). In addition, PFD can also inhibit differentiation of myofibroblasts by regulating PDFG, a fibroblast mitogen receptor, but the specific mechanism is still unclear (Ruwanpura et al., 2020). Studies have found that PFD can resist the loss of E-cadherin, the main intermediary protein of A549 cell epithelial cell transformation induced by TGF-β, and pulmonary fibrosis in a rat model of silicosis, indicating that PFD can also inhibit epithelial cell transformation (Zhang Y et al., 2019). The oxidative stress process in lung diseases leads to irreversible oxidative modification of protein and DNA and mitochondrial dysfunction. PFD treatment can improve mitochondrial respiration, possibly by detoxifying mitochondrial peroxidase, such as glutathione peroxidase, thus revealing ability to maintain normal mitochondrial function (Plataki et al., 2019). Therefore, PFD’s anti-fibrosis effect may function through reducing the formation of reactive oxygen species and oxidative stress.

### Nintedanib

Nintedanib is a triple tyrosine kinase inhibitor with anti-fibrotic effects. In IPF treatment, Nintedanib can reduce the decline of forced vital capacity (FVC) and inhibit progression of pulmonary fibrosis (Flaherty et al., 2019; Makino, 2021). In previous clinical treatments, its safety and tolerability were acceptable. The most common adverse reaction is gastrointestinal infection, manifested as diarrhea and nausea (Bendstrup et al., 2019). Nintedanib can block activation of PDGF receptor, fibroblast growth factor receptor, vascular endothelial growth factor receptor and Src family kinases. Its anti-fibrosis effect is achieved through a variety of mechanisms, including blocking differentiation from fibroblasts into myofibroblasts, inhibiting EMT, inflammation and angiogenesis (Liu F et al., 2019).

### Combination Therapies

As mentioned above, pulmonary fibrosis is a complex pathological progression in which pathogenic factors activate complex fibrotic pathways in various cells (Schafer et al., 2020). Therefore, combined treatment of multiple drugs with different targets and mechanisms involved in IPF is of great significance, but adverse effects and tolerance also need attention. It is currently proven that the combination therapy of pirfenidone and nintedanib has controllable safety and tolerability (Vancheri et al., 2018). Although this study has no efficacy evaluation, it still provides a significant research direction for combination therapy of pulmonary fibrosis.

In a recent clinical phase 2b trial, patients with advanced IPF and pulmonary hypertension were treated with pirfenidone plus sildenafil for up to 52 weeks. Unfortunately, there was no therapeutic effect. (Behr et al., 2021). Furthermore, the combination therapy of nintedanib and sildenafil only has pronounced effects on IPF patients who have right heart dysfunction (RHD) (Behr et al., 2019). Combination treatment with inhaled N-acetylcysteine and pirfenidone for 48 weeks may lead to a worse prognosis of IPF (Sakamoto et al., 2021). These clinical trials indicate that combination therapies for pulmonary fibrosis still need to be explored for a long time.

### Novel Therapies

Due to adverse effects of currently drugs for IPF and there is no effective cure, IPF research is increasingly focused on developing new molecular targets and treatment options. As mentioned above, CTGF is an important pro-fibrotic growth factor associated with extracellular matrix secretion and abnormal tissue repair (Yanagihara et al., 2021). A recent phase II clinical trial has confirmed that treatment of pamrevlumab, which is a fully human recombinant monoclonal antibody against CTGF, can significantly reduce the decline of FVC and attenuate the progression of IPF. And importantly, it is shown that pamrevlumab has good safety and tolerability which is expected to become a new anti-fibrotic drug (Richeldi et al., 2020). In addition, αvβ6 integrin is the key molecule for activating TGF-β. A selective small molecule RGD-mimetic αvβ6 inhibitor GSK3008348, which can bind to αvβ6 with high affinity in human IPF lung epithelial cells, induces αvβ6 internalization and degradation, and inhibits activation of downstream TGF-β. In the bleomycin-induced mouse lung fibrosis model, significantly reduce lung collagen deposition and serum C3M (a marker of IPF disease progression). At present, inhaled GSK3008348 is safe and well tolerated in phase 1 clinical trials, which may be helpful for development of anti-fibrosis drugs in the future (John et al., 2020).

## Conclusion and Outlook

Idiopathic pulmonary fibrosis is an interstitial pulmonary disease with high mortality. It is associated with a large economic and healthcare burden. Genetic and epigenetic changes are important factors in the pathogenesis of IPF, although the definite cause of IPF has yet to be clarified. Our understanding of the pathogenesis of IPF have significantly improved in the past decade. In the last few years, the research progress of idiopathic pulmonary fibrosis has highlighted the important role of stem cell dysfunction and extracellular matrix in mediating lung pathological remodeling and promoting the process of fibrosis (Deng et al., 2020; Wu et al., 2020). Unfortunately, IPF has a very small number of treatments and is still not curable. Nintedanib and pirfenidone can slow the progression of the disease. However, adverse reactions limit their use (Spagnolo et al., 2018). A better understanding of the pathogenesis of IPF and the signal pathways will benefit the development of more effective drug therapies. Gene editing technology provides a promising tool for developing novel treatments for human diseases. CRISPR-mediated genome and epigenome editing may prove to be effective means for correction of abnormal gene expression associated with IPF, thus representing an important direction in the future research. In addition, recent dynamic research activities in IPF pathogenesis have led to the foundation of some novel treatment strategies and identification of therapeutic targets. Several targeted drugs have been further synthesized and developed, and currently under clinical trials. Some of these drugs have been confirmed their effectiveness and safe tolerance. They are expected to become new IPF specific drugs to improve progression of IPF and other fibrosis diseases in the near future.
